# Supplementary material for: Complexome profiling of the Chlamydomonas psb28 mutant reveals TEF5 as an early PSII assembly factor
Source: Plant Cell. 2025 Mar 17;37(6):koaf055. doi: 10.1093/plcell/koaf055 (PMC12164590; doi:10.1093/plcell/koaf055)
Supplement: koaf055_Supplementary_Data [file koaf055_supplementary_data.zip › psb28_interactive.zip!/plots/intensity/qualityPlots/peptides/ANR1_Cre03.g164000.t1.2.html]

### Cre03.g164000.t1.2

Trivial name: ANR1  
SeqCoverage [median]: 51.50  
Mass: 24.23 kDa  
  
 

| Sequence | MissedCleavage | Mass [Da] | wtIntensity | muIntensity | Score |
| --- | --- | --- | --- | --- | --- |
| APAGKAPVYIGFVKEEGFGNR | 2 | 2206.15 | 2116.47 | 0.00 | 75.24 |
| APVYIGFVK | 0 | 992.57 | 23986.49 | 127380.50 | 148.07 |
| APVYIGFVKEEGFGNR | 1 | 1781.91 | 55832.59 | 125591.55 | 134.24 |
| APVYIGFVKEEGFGNRDGK | 2 | 2082.05 | 6268.21 | 7365.95 | 95.35 |
| APVYIGFVKEEGFGNRDGKR | 3 | 2238.15 | 3918.88 | 3865.75 | 70.96 |
| DSKKPAAPAPSSPLPVK | 2 | 1688.95 | 2392.37 | 1307.26 | 50.05 |
| DSKKPAAPAPSSPLPVKVPK | 3 | 2013.16 | 5092.12 | 0.00 | 55.33 |
| EDLGPLPGVTGGFAAGEVGLK | 0 | 1983.03 | 451.37 | 0.00 | 56.29 |
| EEGFGNR | 0 | 807.35 | 1975.25 | 22123.51 | 144.29 |
| EEGFGSR | 0 | 780.34 | 579.71 | 10054.97 | 85.36 |
| EQVIKEK | 1 | 872.50 | 362.12 | 0.00 | 125.25 |
| EQVIKEKDSK | 2 | 1202.65 | 2844.95 | 1769.72 | 124.67 |
| GRIVVDDPRK | 2 | 1153.66 | 862.94 | 1641.71 | 58.37 |
| IVVDDPR | 0 | 812.44 | 22866.22 | 29119.74 | 108.24 |
| IVVDDPRK | 1 | 940.53 | 19842.47 | 29691.36 | 112.17 |
| KPAAPAPSSPLPVK | 1 | 1358.79 | 23145.68 | 107968.56 | 146.67 |
| KPAAPAPSSPLPVKVPK | 2 | 1683.01 | 36909.76 | 21396.10 | 116.86 |
| KYGGKEDLGPLPGVTGGFAAGEVGLK | 2 | 2516.33 | 1753.48 | 5896.21 | 72.23 |
| LRQPGQPGPR | 1 | 1104.62 | 92897.69 | 133961.17 | 149.27 |
| QPGQPGPR | 0 | 835.43 | 0.00 | 6297.43 | 104.76 |
| QYVATGDVK | 0 | 979.50 | 63962.97 | 74860.53 | 177.91 |
| VIKDDPK | 1 | 813.46 | 0.00 | 7561.18 | 124.34 |
| YGGKEDLGPLPGVTGGFAAGEVGLK | 1 | 2388.23 | 13325.78 | 26247.52 | 131.35 |
|  |  |  | 381387.5 | 744100.7 |  |
